# The reliability of non-invasive neurological examinations in people with diabetes

**DOI:** 10.1186/1757-1146-6-S1-O6

**Published:** 2013-05-31

**Authors:** Sean Lanting, Peta Craike, Martin Spink, Sarah Casey, Vivienne Chuter

**Affiliations:** 1 Podiatry Discipline, University of Newcastle, Ourimbah, NSW, 2258, Australia

## Background

Pinprick perception, 128-Hz tuning fork vibration detection, ankle reflexes, vibration perception threshold (VPT) with a biothesiometer or similar instrument, and four site 10g monofilament assessment are all currently recommended methods for screening for diabetic peripheral neuropathy (DPN). However, there is limited research investigating the reliability of these tests. The aim of this study was to determine the inter- and intra-tester reliability of the five neurological tests currently recommended for screening for DPN.

## Methods

All five recommended neurological examinations were performed by three clinicians on people with diabetes to determine inter-tester reliability. The tests were repeated by the same clinicians seven days later to determine intra-tester reliability.

## Results

Fifty participants with diabetes were recruited to this study, 44 returned for the re-test. The intra-tester reliability of the VPT with the neurothesiometer was substantial (К: 0.52-0.78), monofilament test was moderate (К: 0.34-0.67), and the pinprick and ankle reflex examinations was fair (К: 0.15-0.32 and 0.09-0.62 respectively). The inter-tester reliability of the neurothesiometer and monofilament was substantial (К: 0.61 respectively), the pinprick examination moderate (К: 0.52) and ankle reflex examination slight (К: 0.12). The reliability of the vibration perception examination using the 128-Hz tuning fork could not be calculated due to all participants recording an abnormal result.

## Conclusion

For the purposes of clinical screening and ongoing monitoring of DPN the four site 10g monofilament test and the vibration perception threshold examination using the neurothesiometer are the most reliable of the recommended screening examination for people with diabetic peripheral neuropathy (DPN).

